# The blood pressure targets in sepsis patients with acute kidney injury: An observational cohort study of multiple ICUs

**DOI:** 10.3389/fimmu.2022.1060612

**Published:** 2022-12-15

**Authors:** Lina Zhao, Yan Fan, Zhiwei Wang, Zhiyong Wei, Ying Zhang, Yun Li, Keliang Xie

**Affiliations:** ^1^ Department of Critical Care Medicine, Tianjin Medical University General Hospital, Tianjin, China; ^2^ Department of Anesthesiology, Tianjin Medical University General Hospital, Tianjin, China; ^3^ Department of Anesthesiology, Tianjin Institute of Anesthesiology, Tianjin, China

**Keywords:** sepsis, mean arterial pressure, diastolic blood pressure, acute kidney injury, hemodynamics

## Abstract

**Background:**

The maintenance of blood pressure is pivotal in preventing sepsis with acute kidney injury (AKI). Especially in sepsis patients treated with vasopressors. The optimal the blood pressure has been controversial to maintain renal perfusion. This study aims to explore the blood pressure target in sepsis with AKI.

**Methods:**

We retrieved patient data from the MIMIC IV and eICU databases. The Lasso regression model was used to identify the relationship between blood pressure and sepsis in patients with AKI and remove collinearity among variables. Generalized additive models were used to estimate the blood pressure range in patients with sepsis with AKI. Statistical methods such as multivariable logistic regression, propensity score analysis, inversion probability-weighting, and doubly robust model estimation were used to verify the target blood pressure for patients with sepsis and AKI.

**Results:**

In total, 17874 patients with sepsis were included in this study. the incidence of AKI may be related to the level of mean article pressure (MAP) and diastolic blood pressure (DBP) in sepsis patients. The range of MAPs and DBPs may be 65-73 mmHg and 50-60 mmHg in AKI patients without hypertension. The range of MAPs and DBPs may be 70-80 mmHg and 54-62 mmHg in AKI patients with hypertension. The prognosis of sepsis with AKI was unaffected by MAP or DBP. Systolic blood pressure is not associated with sepsis in patients with AKI.

**Conclusions:**

To ensure renal perfusion, AKI patients with hypertension may require a higher MAP [70-80] versus (65-73), mmHg] and DBP [(54-62) vs (50-60), mmHg] than patients without hypertension.

## Introduction

Acute kidney injury (AKI) has been a global concern in the field of acute and critical diseases (1). According to a multinational cross-sectional survey, over 50% of intensive care unit (ICU) patients suffer from AKI, with hospital mortality being linked to severity (2). The incidence of AKI is high, and it is associated with short-term as well as long-term mortality among severe patients especially patients with sepsis and shock. In the ICU, sepsis with AKI develops in 50%-70% of patients and acts as an independent risk factor for mortality in hospitalized patients (3, 4). AKI patients with Sepsis usually develop more severe symptoms with higher mortalities, resulting in a significant economic burden on patients, families, and society. This is an urgent clinical problem that needs to be resolved in the field of acute and severe diseases (5).

Despite receiving adequate fluid resuscitation and vasopressors, many patients with sepsis still suffer from organ hypoperfusion. Insufficient renal perfusion and low blood pressure are health risks that contribute to AKI. The Surviving Sepsis Campaign recommends that patients with low blood pressure receive vasopressors to raise their blood pressure (6) where lactate levels were used to determine this recommendation. Maintaining renal perfusion in sepsis requires higher MAP (>75 mmHg) levels, according to Martin Dünser et al. (7). According to a multicenter randomized controlled trial (RCT) study focusing on sepsis patients with AKI prognosis, patients were divided into two groups according to MAP level, with high MAP levels of 80 to 85 mmHg, and low MAP levels of 65 to 70 mmHg whose results showed that AKI patients do not have any difference in prognosis. Patients with AKI and chronic hypertension who had high MAP levels were less likely to develop AKI and required fewer CRRT treatments than those with low MAP levels (8). Saito et al. reported measurement of ICU hemodynamic parameters including systolic blood pressure (SBP), DBP, MAP, and central venous pressure while calculating the mean perfusion pressure as well as diastolic perfusion pressure according to these hemodynamics parameters where they found that there was no difference in the percentage of SBP or MAP between AKI+ and AKI-. The DBP, diastolic perfusion pressure, and mean perfusion pressure, however, showed significant variations (9). An MAP that maintains renal perfusion remains controversial. Hemodynamic management of sepsis with AKI has always been a hot topic of discussion (10).

The effects of MAP on renal injury have been studied through RCTs, but the effects of SBP and DBP have been poorly investigated. The effects of SBP and DBP have been studied in a small sample size but the impact of extremely high blood pressure on patients has rarely been considered. Thus, there are still no a large-scale study with comprehensive blood pressure assessments in patients with sepsis and AKI. The effect of blood pressure on kidney is still a hot topic and controversial. Sepsis patients with AKI with chronic hypertension may and without hypertension need different blood pressure levels to maintain kidney function. As a result of the above study, sepsis patients with AKI were categorized into patients with chronic hypertension and those without chronic hypertension. Through large multiple databases, we explored the blood pressure of sepsis patients with AKI who had hypertension and those who did not have hypertension using the incidence of AKI as the main research result, and atrial fibrillation as an adverse event caused by high blood pressure.

## Materials and methods

### Study settings

This study was a large observational study from the multicenter database eICU Collaborative Research Database (eICU-CRD v2.0) from 2014 to 2015 and Medical Information Mart for Intensive Care IV (MIMIC-IV version 1.0) database from 2008 to 2019 (11, 12). The author of this study has completed the collaborative institutional training initiative examination (certification number 33690380) and can access the database. They all have passed the review of the ethics committee.

### Patients

This study population conforms to the diagnostic criteria of sepsis 3.0 (13). In this study, sepsis was defined as a suspected infection in conjunction with an acute increase in the Sequential Organ Failure Assessment (SOFA) score ≥ 2. If the patient was suspected of having an infection or was prescribed antibiotics, bodily fluids were sampled for microbiological culture. After the antibiotic is administered, a microbiological sample must be obtained within 24 hours; after the microbiological sample is collected, the antibiotic must be administered within 72 hours. In this study, sepsis patients with AKI were included during the hospitalizationg, and AKI was defined according to the Kidney Disease Improving Global Outcomes (KDIGO) criteria (14). This study focused on adult patients (aged >18) who stayed in the intensive care unit for more than 48 hours. Sepsis patients without vasopressors drugs in the period of hospitalization, missing blood pressure values were excluded from the study. Furthermore, this study looked for patients with atrial fibrillation in sepsis based on previous studies that showed a high MAP led to atrial fibrillation. A secondary diagnosis of atrial fibrillation was made during hospitalization, and the patient was treated with antiarrhythmic drugs. Patients with atrial fibrillation who meet the above conditions are considered atrial fibrillation.

### Data collection

We collected patient age, gender, coexisting illnesses, infection site, and microbiological infection type data. During hospitalization and treatment with vasopressor drugs, the mean value of vital signs and urine output as well as the worst laboratory parameters were recorded. Patient’s disease severity score, including SOFA and GCS. In addition to recording if the patients were treated with mechanical ventilation, the patients’ length of stay, length of stay in the ICU, and their hospital mortality were also recorded. Only the first admission was considered for patients who are admitted to the ICU repeatedly.

### Statistical analysis

The Shapiro Wilk test was used in this study to detect distributions of data. This study uses continuous variables with non-normal distributions. Several continuous variables are described by the median and interquartile range (IQR). There are also categorical variables that are expressed as a count and a percentage. The two groups of continuous variables were compared using a nonparametric test. The categorical variables were compared using Fisher’s exact test.

To reduce multicollinearity between variables, the Lasso regression model was used to select variables that were significantly different from each other in **Table 1** (15). To determine which blood pressure range is most for different AKI populations in terms of the incidence of AKI and atrial fibrillation, the generalized additive model was used to estimate the range of blood pressure-related variables selected by the lasso regression model (16).

**Table 1:** Baseline characteristics and outcomes of patients with sepsis.

	Non-AKI patients (n = 12041)	AKI patients (n = 5833)	P
Baseline variables			
Age(years) (median [IQR])	68.00 [58.00, 77.00]	71.00 [61.00, 80.00]	<0.001
Gender, M (%)	5136 ( 42.7)	2256 (38.7)	<0.001
Coexisting illness, (n(%))			
Hypertension	2212 ( 18.4)	1587 (27.2)	<0.001
Diabetes	2123 ( 17.6)	1850 (31.7)	<0.001
Chronic lung disease	1494 ( 12.4)	1218 (20.9)	<0.001
Cardiovascular disease	3430 (28.5)	2683 (46.0)	<0.001
Site of infection, (n (%))			
Urinary	1284 ( 10.7)	855 (14.7)	<0.001
Lung	996 ( 8.3)	653 (11.2)	<0.001
Catheter	109 (0.9)	206 (3.5)	<0.001
Skin soft tissue	668 (5.5)	434 (7.4)	<0.001
Abdominal cavity	490 ( 4.1)	373 (6.4)	<0.001
Microbiology type, (n (%))			
Acinetobacter baumannii	7 (0.1)	32 ( 0.5)	<0.001
Klebslella	191 (1.6)	571 (9.8)	<0.001
Escherichia Coli	453 (3.8)	959 (16.4)	<0.001
Pseudomonas aeruginosa	120 ( 1.0)	361 (6.2)	<0.001
Staphylococcus aureus	1118 ( 9.3)	1617 (27.7)	<0.001
Fungus	399 ( 3.3)	1355 (23.2)	<0.001
Vital signs, (median [IQR])			
Heart rate(bpm)	93.00 [83.00, 106.00]	100.00 [87.00, 116.00]	<0.001
Respiratory rate (bpm)	23.00 [20.00, 28.00]	27.00 [22.00, 32.00]	<0.001
Systolic blood pressure (mmHg)	96.00 [85.00, 113.00]	88.00 [78.00, 100.00]	<0.001
Diastolic blood pressure (mmHg)	53.00 [45.00, 62.00]	47.00 [40.00, 55.00]	<0.001
Mean arterial pressure(mmHg)	70.00 [60.00, 84.00]	58.00 [52.00, 67.00]	<0.001
Laboratory parameters (median [IQR])			
White blood cell (×10^9^ /L)	15.30 [11.00, 20.60]	14.30 [10.00, 20.10]	<0.001
Hemoglobin(g/dL)	9.50 [8.20, 10.90]	9.00 [7.80, 10.60]	<0.001
Platelet (×10^9^ /L)	153.00 [109.00, 213.00]	152.00 [102.00, 223.00]	0.217
Creatinine (mg/dL)	1.10 [0.80, 2.00]	1.80 [1.20, 3.00]	<0.001
Blood urea nitrogen (mg/dL)	23.00 [15.00, 40.00]	36.00 [23.00, 58.00]	<0.001
Glucose(mg/dL)	140.00 [115.00, 186.00]	152.00 [121.00, 210.00]	<0.001
Sodium (mmol/L)	138.00 [135.00, 141.00]	139.00 [135.00, 142.00]	<0.001
Potassium(mmol/L)	4.50 [4.10, 5.00]	4.70 [4.20, 5.60]	<0.001
Lactates (mmol/L)	2.30 [1.50, 3.90]	2.00 [1.30, 3.50]	<0.001
Urine output(mL)	611.00 [150.00, 1757.00]	1012.00 [325.00, 1760.00]	<0.001
The score system, (median [IQR])			
SOFA	6.00 [4.00, 9.00]	7.00 [5.00, 10.00]	<0.001
GCS	12.00 [12.00, 14.00]	13.00 [10.00, 15.00]	0.009
Mechanical ventilation, (n(%))	5959 ( 49.5)	4137 ( 70.9)	<0.001
Outcome			
Atrial fibrillation, (n (%))	4086 ( 33.9)	1742 ( 29.9)	<0.001
RRT, (n (%))	1412 ( 11.7)	1211 ( 20.8)	<0.001
Length of ICU stays, days (median [IQR])	2.80 [1.37, 5.60]	3.40 [1.80, 7.73]	<0.001
Length of hospital stays, days (median [IQR])	8.00 [5.10, 14.20]	11.60 [6.40, 20.70]	<0.001
Hospital mortality, (n (%))	2353 ( 19.5)	1625 ( 27.9)	<0.001

GCS, Glasgow coma scale; SOFA, sequential organ failure assessment; RRT, renal replacement therapy; ICU, intensive care unit.

We tested the relationship between blood pressure and AKI patients using a multivariate Logistic Regression model. An independent association between optimal blood pressure levels and patients’ AKI was inferred through the doubly robust estimation method (17). Multivariate Logistic regression and Extreme Gradient Boosting (XGBoost) were used to create propensity score models for the 29 covariables in sepsis patients with AKI and chronic hypertension. A cohort of inverse probability of treatment weighting (IPTW) was generated from the estimated propensity scores (18). Afterward, we performed a Logistic Regression on the weighted cohort to adjust for remaining unbalanced variables in the propensity score model between AKI groups and non-AKI groups, resulting in a double robust analysis. To determine whether IPTW reduced the imbalance of covariate distribution, the standardized mean difference (SMD) of the original cohort was compared with the SMD of the IPTW cohort. R software was used to carry out all statistical analyses, and P <0.05 is considered statistically significant.

## Results

### Baseline characteristics

**Figure 1:**
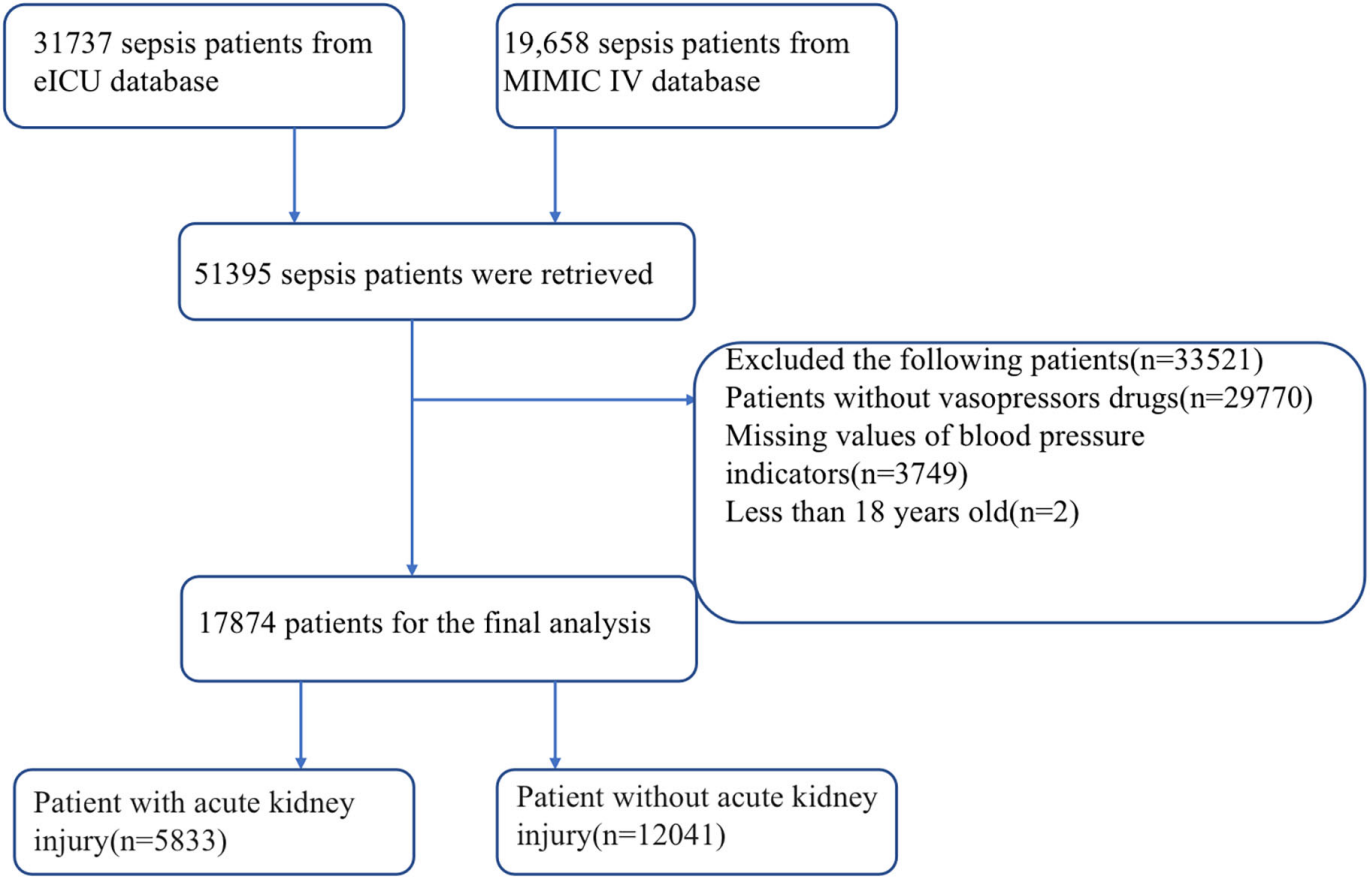
Flow chart for patient selection. ICU, intensive care unit; MIMIC-IV, Medical Information Mart for Intensive Care IV.

A total of 51395 sepsis patients were retrieved from MIMIC IV and the eICU databases. Of these, 33521 patients were excluded based on the exclusion criteria. A total of 17874 patients were included in the study. The number of patients with Sepsis with AKI was 5833 while the number of patients with sepsis without AKI was 12041 (**Figure 1**).

**Table 1** are described the baseline characteristics of the patients. The incidence of multiple site infections and multiple microbiology was higher in patients with AKI compared with sepsis patients without AKI whereas the level of sodium, potassium, glucose, hemoglobin, and blood urea nitrogen was worse in patients with AKI. Compared to sepsis patients without AKI, sepsis patients with AKI had higher SOFA scores, higher rates of mechanical ventilation and RRT, longer hospital stays, ICU stays, and higher hospital mortality (**Table 1**).

### Characteristic variable for incidence of AKI

**Figure 2:**
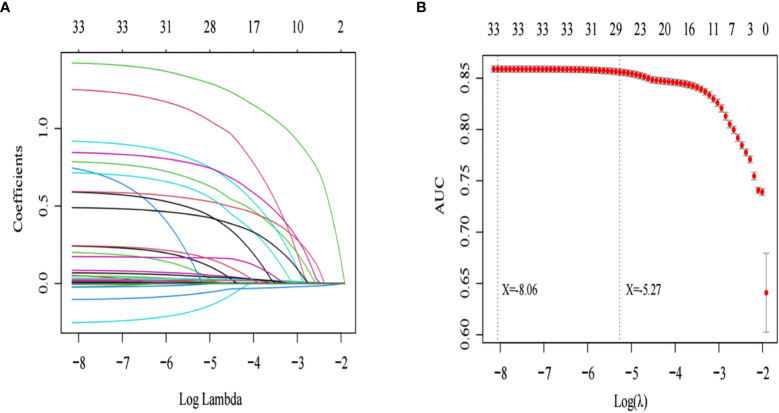
Lasso regression was used to screen the characteristic variables. **(A)** shows that with the increase of log lambda value, the punishment to the model increases, and fewer characteristic variables are included in the model. The dotted line on the left of **(B)** indicates the inclusion of the minimum model independent variables, the dotted line on the right indicates the inclusion of independent variables in the most concise model.

In **Table 1** the results show that there are differences in many variables between sepsis patients with AKI and without AKI. The patients’ diseases are very serious, and many significant differences variables are likely to have collinearity. To remove collinearity between the variables, we used the Lasso regression model to screen the significantly different variables. As shown in **Figure 2B**, two models are obtained after removing the existing collinearity variable. The dotted line at the left represents the minimum model, which contains 33 variables [Log(λ): -8.06]. As shown in **Figure 2A**, the dotted line on the right represents the streamlined model, which contains 29 variables [Log(λ): -5.27].

### Generalized additive models to estimate the blood pressure targets for incidence of AKI

**Figure 3:**
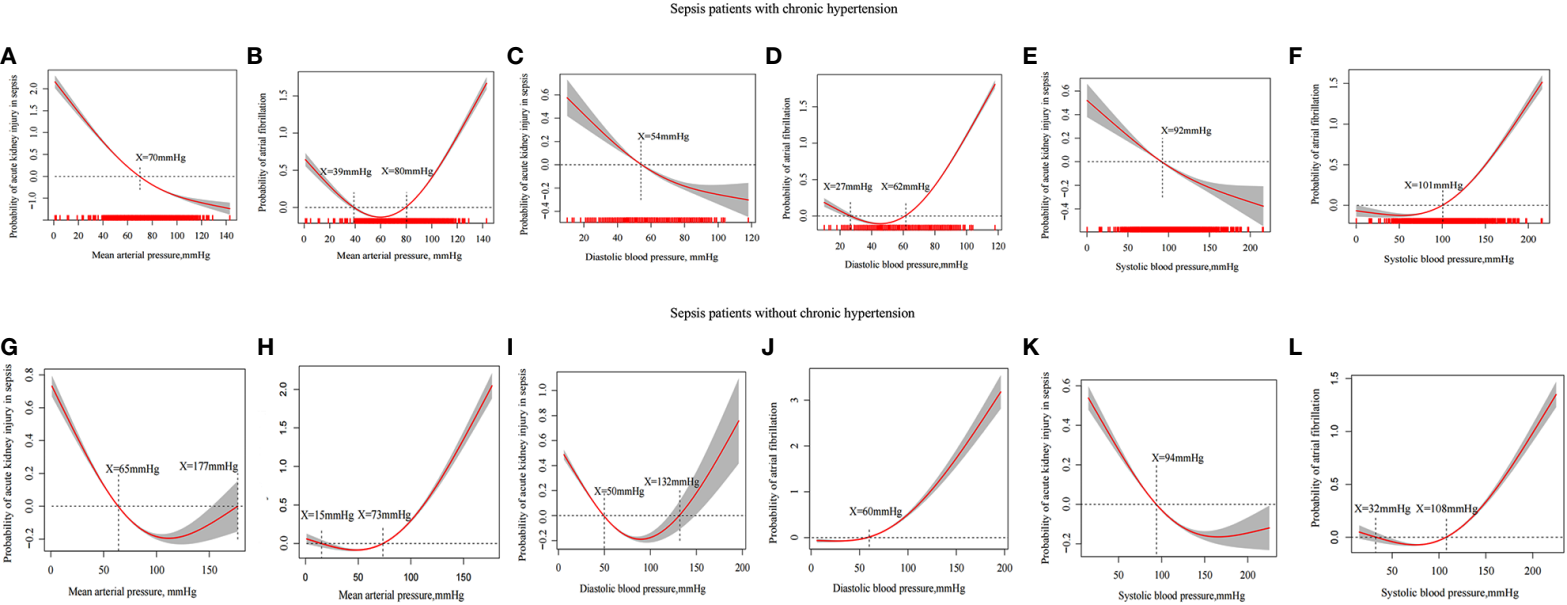
Generalized additive model evaluates the relationship between MAP, DBP, SBP and incidence in sepsis patients with AKI and incidence of atrial fibrillation. The relationship between MAP **(A)**, DBP **(C)** and SBP **(E)** and the incidence of AKI in sepsis patients with hypertension. The relationship between MAP **(G)**, DBP **(I)** and SBP **(K)** and the incidence of AKI in sepsis patients without hypertension. The relationship between MAP **(B)**, DBP **(D)** and SBP **(F)** and the incidence of atrial fibrillation in sepsis patients with chronic hypertension. The relationship between MAP **(H)**, DBP **(J)** and SBP **(L)** and the incidence of atrial fibrillation in sepsis patients with chronic hypertension.

We divided the patients into two groups according to whether they had hypertension so that we could evaluate the SBP, DBP, and MAP of the incidence of AKI using a generalized additive model. According to the results of the study, MAP≥70 mmHg (**Figure 3A**), DBP≥54 mmHg (**Figure 3C**) and SBP≥92 mmHg (**Figure 3E**) reduced the incidence of AKI among sepsis patients with hypertension. In patients with sepsis without chronic hypertension, this study showed a nonlinear relationship between DBP, MAP, and the incidence of AKI. For AKI incidence (P<0.001), the MAP ranged from 65 to 177 mm Hg (**Figure 3G**), DBP was 50 to 132mmHg (**Figure 3I**) and SBP was more than 94mmHg (**Figure 3K**).

A high MAP was linked to atrial fibrillation in previous RCT studies. A high MAP, DBP, and SBP of patients were limited in the above population when atrial fibrillation incidence is taken as the endpoint. There was an increased incidence of atrial fibrillation in patients having sepsis with chronic hypertension who had MAP ≥80mmHg (**Figure 3B**), DBP ≥62mmHg (**Figure 3D**), and SBP≥101mmHg (**Figure 3F**) whereas there was an increased incidence of atrial fibrillation in patients with sepsis without chronic hypertension who had MAP ≥73 mmHg (**Figure 3H**), DBP ≥60 mmHg (**Figure 3J**), and SBP ≥108 mmHg (**Figure 3L**).

### Multivariate logistic analysis for incidence of AKI in sepsis patients

According to the results of the generalized additive model, the range of MAP, DBP, and SBP of AKI patients with hypertension was (70-80) mmHg, (54-62), and (92-101) mmHg, respectively. In patients with AKI without hypertension disease, the MAP, DBP, and SBP ranges (65-73) mmHg, (50-60) mmHg, and (94-108) mmHg, respectively. Moreover, we selected variables that were contained in the most streamlined model screened by Lasso regression for multivariate analysis (**Supplementary Material 1**). The results of **Table 2** show that MAP (70-80) mmHg [OR: 0.60, 95% CI: 0.45-0.80, P<0.001], DBP (54-62) mmHg [OR: 0.65, 95% CI: 0.54-0.77, P<0.001] were independent protective factors in sepsis patients with AKI with chronic hypertension. It was found that the MAP (65-73) mmHg [OR: 0.82, 95% CI: 0.72-0.93, P=0.033], and the DBP (50-60) mmHg [OR: 0.89, 95% CI: 0.77-0.99, P=0.038] were independent protective factors in sepsis patients with AK without chronic hypertension. SBP is not an independent protective factor for septic AKI (**Table 2**).

**Table 2:** Application of multiple models to explore blood pressure indicators to predict the occurrence of sepsis with AKI.

Models	OR	CI		P
		2.5%	97.5%	
**Patients without hypertension disease**				
**Lasso regression + Multivariate Logistic analysis**				
Mean arterial pressure (65-73) mmHg	0.82	0.72	0.93	0.003
Diastolic blood pressure (50-60) mmHg	0.89	0.78	0.99	0.038
SBP(94-108)mmHg	0.91	0.82	1.02	0.101
**Propensity score matching**				
Mean arterial pressure (65-73) mmHg	0.88	0.80	0.97	0.008
Diastolic blood pressure (50-60) mmHg	0.73	0.66	0.79	<0.001
SBP(94-108)mmHg	0.83	0.76	0.91	<0.001
**Propensity score IPTW**				
Mean arterial pressure (65-73) mmHg	0.83	0.75	0.93	0.001
Diastolic blood pressure (50-60) mmHg	0.85	0.77	0.94	0.002
SBP(94-108)mmHg	0.96	0.87	1.07	0.472
**Doubly robust with all covariates**				
Mean arterial pressure (65-73) mmHg	0.84	0.77	0.91	<0.001
Diastolic blood pressure (50-60) mmHg	0.89	0.82	0.95	0.001
SBP(94-108)mmHg	0.94	0.87	1.02	0.133
**Patients with hypertension disease**				
**Lasso regression + Multivariate Logistic analysis**				
Mean arterial pressure (70-80) mmHg	0.60	0.45	0.80	<0.001
Diastolic blood pressure (54-62) mmHg	0.65	0.54	0.77	<0.001
SBP(92-101)mmHg	0.99	0.80	1.23	0.959
**Propensity score matching**				
Mean arterial pressure (70-80) mmHg	0.72	0.55	0.94	0.018
Diastolic blood pressure (54-62) mmHg	0.82	0.68	0.98	0.028
SBP(92-101)mmHg	0.96	0.77	1.12	0.729
**Propensity score IPTW**				
Mean arterial pressure (70-80) mmHg	0.57	0.44	0.73	<0.001
Diastolic blood pressure (54-62) mmHg	0.54	0.46	0.63	<0.001
SBP(92-101)mmHg	0.93	0.76	1.13	0.456
**Doubly robust with all covariates**				
Mean arterial pressure (70-80) mmHg	0.76	0.63	0.91	0.004
Diastolic blood pressure (54-62) mmHg	0.73	0.66	0.80	<0.001
SBP(92-101)mmHg	0.97	0.86	1.10	0.643

### Propensity match analysis

**Figure 4:**
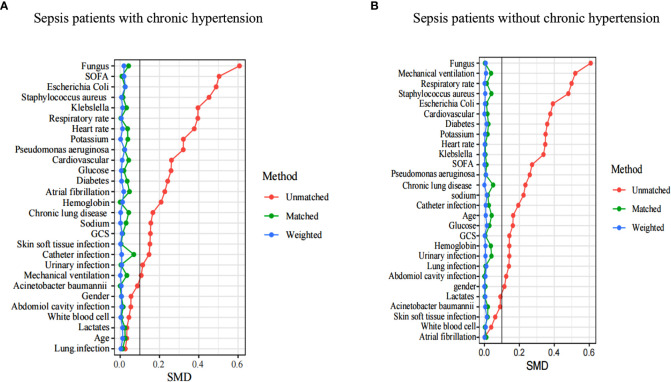
A SMD of the original cohort was compared with the SMD of the IPTW cohorts. In sepsis AKI incidence, sepsis patients with hypertension **(A)** and sepsis patients without hypertension **(B)** showed that covariates were well balanced between classes after IPTW(<0.1). SMD, standardized mean difference, IPTW, inverse probability of treatment weighting .

In terms of AKI incidence, the double-robust analysis showed that MAP, DBP, and SBP had a significant beneficial effect. A propensity matching scoring model was constructed using 29 covariates with statistically significant differences in **Table 1** except platelets, creatinine, blood urea nitrogen, urine output, RRT, length of ICU stays, length of hospital stays, ICU mortality, systolic blood pressure, diastolic blood pressure, and mean arterial pressure. For standardizing the differences between the AKI group and the non-AKI group, the estimated propensity scores were used. Covariates were well balanced between classes after IPTW (<0.1) (**Figure 4**). To evaluate the relationship between the MAP, DBP, and SBP levels (estimated as per generalized additive model) and AKI incidence, we used four different models: statistical analysis, propensity matching score, proportion score IPTW and doubly robust model. The estimation models led to the same conclusion: MAP (70-80) mmHg and DBP (54-62) mmHg were protective factors for patients with AKI with hypertension disease; MAP (65-73) mmHg and DBP (50-60) mmHg are protective factors of patients with AKI without hypertension disease (**Table 2**).

### Prognostic analysis of blood pressure and sepsis patients with AKI

In septic patients with AKI without chronic hypertension, MAP (65-73) mmHg and DBP (50-60) mmHg were associated with less atrial fibrillation and lower creatinine and blood urea nitrogen levels; whereas, among sepsis patients having AKI with chronic hypertension, MAP (70-80) mmHg and DBP (54-62) mmHg were linked to less atrial fibrillation, lower creatinine, and lower urea levels. There was no association with length of hospital stays, ICU stays, and hospital mortality in patients with sepsis AKI (**Supplementary Material 2**).

## Discussion

The incidence of AKI was 32.63%. Sepsis patients with AKI had poor clinical outcomes. The range of MAP and DBP may be (65-73) mmHg and (50-60) mmHg in sepsis patients with AKI who did not have hypertension. The range of the MAP and DBP may be (70-80) mmHg and (54-62) mmHg in sepsis patients with AKI having hypertension. It was found that MAP and DBP were not linked with the prognosis of sepsis patients with AKI.

Sepsis with AKI has always been a disease of global concern, it has a high incidence rate and is associated with poor clinical outcomes. Sean m Bagshaw et al. found that the incidence of AKI was 64.4% and AKI was mainly associated with significant ICU mortality and hospital mortality (OR 1.73 and OR 1.62), respectively (3). A Korean population cohort study found that patients with AKI had higher hospital mortality, longer ICU stays, and higher total costs (4). This study is based on a large multicenter cohort study that indicated that the incidence of AKI was 32.63%, and sepsis patients with AKI had higher SOFA scores, more patients use mechanical ventilation, longer hospital stay, ICU stays, and shown higher hospital mortality as compared to sepsis patients without AKI. The results of this study are consistent with similar studies conducted in the past. However, the incidence of septic AKI in this study is found to be lower than that of the investigation performed by Bagshaw et al. This difference may be attributed to the use of different AKI diagnostic methods. In this study, KDIGO criteria were adopted, while Bagshaw et al. adopted RIFLE criteria. Recent research by Zhang and coworkers found that the incidence of AKI in sepsis was 41.1% in the mimic IV database (19), the results of this study support the findings of Luming Zhang and other colleges. The above studies indicate that patients with Sepsis AKI are still diseases that require urgent attention in critical care medicine.

Most sepsis patients are known to have low blood pressure and insufficient tissue perfusion. These two parameters (low blood pressure levels and renal hypoperfusion) are the important mechanisms of AKI in sepsis (20). It has always been a clinical hot topic to maintain blood pressure levels of renal perfusion, but it is still controversial. Martin WD ü nser et al. proposed that MAP > 75 mmHg may be sufficient to maintain kidney function (7). The study made a better proposal for maintaining kidney function, however, it did not consider whether the patients had chronic hypertension or the adverse effect caused by patients with high MAP. Pierre asfar et al. conducted an RCT study after the study of Martin W D ü nser et al. Pierre asfar et al. made up for the study limitation of Martin W D ü nser et al. in sepsis AKI, they considered that AKI patients with chronic hypertension may need higher MAP levels and the harm that caused by higher MAP to the patient’s body. Asfar et al. suggested that patients suffering from chronic hypertension, target a MAP of 80 to 85 mm Hg, and patients without a history of chronic hypertension, target a MAP of 65 to 70 mm Hg. It was an observation that higher MAP did not have any significant impact on the prognosis of AKI patients; however, in addition, it will enhance the incidence of atrial fibrillation (8). The study led by Asfar et al. provides strong evidence for the control of MAP levels among patients with sepsis AKI. However, in the same study, the mortality of AKI patients was the primary outcome with an exploration of the MAP level, not AKI incidence, which may deviate from the MAP level for the incidence of AKI in sepsis. Besides, the study only considered MAP and did not consider the impact of SBP and DBP levels on AKI patients. The study also classifies the high MAP group (80-85) mmHg and the low MAP group (65-70) mmHg on the bases of clinical observation, there may be bias in the accurate MAP level. In another study by Forni and coworkers, the impact of MAP on AKI was questioned and discussed. They provided the detailed suggestion that why one should not only pay attention to MAP, DBP, renal systolic perfusion pressure and diastolic perfusion pressure, but other indicators also play a key role which should also be given proper attention (20, 21). Based on the results of previous studies, in this study, AKI incidence and atrial fibrillation as an outcome were explored for levels of SBP, DBP, and sepsis MAP in patients with AKI. The blood pressure level was estimated by a generalized additive model rather than by dividing by clinical experience. In many articles, the generalized additive model is used in evaluating the index level  (22, 23).

The surprising finding of this study was that there was a nonlinear relationship between MAP, DBP, and the incidence of sepsis in patients with AKI without chronic hypertension. They did, however, have a linear relationship with sepsis patients with AKI with chronic hypertension. It may be attributed to the increase of anterior glomerular arteriole resistance and intraglomerular hypertension and the continuous increase of anterior glomerular artery resistance which brings the glomerular capillaries into a state of high perfusion, high filtration, and high transmembrane pressure and ultimately in the state of long-term chronic hypertension (24). Glomerular capillaries are in a state of high perfusion and high filtration for a long time, which can withstand the level of high blood pressure in sepsis. MAP > 177 mmHg and DBP > 132 mmHg will cause an increase in glomerular pressure leading to kidney damage in sepsis patients without chronic hypertension. Furthermore, MAP < 65 mmHg and DBP <50 mmHg can lead to insufficient renal perfusion and renal injury in sepsis patients without chronic hypertension. We need to control the patient’s MAP ≥ 65 mmHg and DBP ≥ 50 mmHg to maintain the patient’s renal perfusion in the condition of sepsis without chronic hypertension. MAP ≥ 65 mmHg is almost in line with the guidelines of the surviving sepsis campaign, expert opinion of the working group on prevention, AKI section, European Society of intensive care medicine (25-27). Sepsis patients with hypertension need to maintain a higher MAP (≥ 70 mmHg) and DBP (≥54 mmHg), which is basically in line with the study conducted by Pierre as far et al, which says that higher MAP can lead to atrial fibrillation. This study shows that MAP>73 mmHg and DBP>60 mmHg can lead to atrial fibrillation in sepsis patients without chronic hypertension, and MAP>80 mmHg and DBP>62 mmHg can lead to atrial fibrillation in sepsis patients having chronic hypertension. In summary, we suggest that the MAP range is 65 to 73 mmHg and the DBP range is 50 to 60 mmHg in sepsis patients with AKI without chronic hypertension; the MAP range is 70 to 80 mmHg and the DBP range is 54 to 62 mmHg in sepsis patients having AKI along with chronic hypertension (**Table 2**). Patients with blood pressure within the range had significantly lower levels of creatinine and blood urea nitrogen than those with AKI outside the range (**Supplementary Material 3**). Unfortunately, this study found that the blood pressure range level was not associated with the prognosis of patients with AKI. This study not only provides the range of MAP but also provides the range of DBP too. It also established that DBP may play an important role in the occurrence of AKI. We have found results that are potentially impactful and that support the resulting study of Pierre et al. However, the highest value of the range of MAP level is higher than Pierre et al. suggested MAP≤ 70 mmHg in sepsis without chronic hypertension, and MAP range is lower than Pierre et al. suggested MAP from 80 to 85 mmHg. The difference could be explained by the different methods of estimating MAP ranges and the different primary outcomes of the studies. Another difference can be as Pierre et al. studied the mortality of sepsis patients with AKI, and we studied the incidence of sepsis in patients with AKI. Although our findings support the blood pressure target for sepsis patients with AKI, the target would not apply in some instances, for example, in patients with severe shock and disturbance of consciousness who may need higher blood pressure levels to maintain their consciousness. Severe shock cannot be corrected and is life-threatening. In addition, our results do not extend to patients who do not take vasopressor drugs.

Through a large observational study, we provide a reference range for blood pressure levels, but it is important to demonstrate the limitations of such studies. First of all, since this is a retrospective study, our results could not provide a causal relationship between the blood pressure level and AKI incidence in sepsis. Secondly, based on previous studies, we chose atrial fibrillation caused by high blood pressure to be the endpoint of blood pressure level control, which may have caused a deviation in the research results. Thirdly, the dose and type of the vasopressors, and the amount of fluid used for residence, were not included in this study, which are important for AKI of sepsis patients, their absence may cause information bias to the study results. We provide a certain reference range for the blood pressure level control of AKI patients based on large-scale data, but some patients don’t fit into this range.

## Conclusion

Based on previous studies, we reassessed the blood pressure range in sepsis patients with AKI using multiple large databases. Through this study, we recommend that the MAP range may be 65 to 73 mmHg and the DBP range may be 50 to 60mmHg in sepsis patients with AKI without chronic hypertension; however, the MAP range may be 70 to 80 mmHg and the DBP range may be 54 to 62 mmHg in sepsis patients with AKI with chronic hypertension.

## Data availability statement

The original contributions presented in the study are included in the article/**Supplementary Material**. Further inquiries can be directed to the corresponding authors.

## Ethics statement

The studies involving human participants were reviewed and approved by MIMIC-IV and eICU-CRD databases were approved by the institutional review boards of the Massachusetts Institute of Technology and Beth Israel Deaconess Medical Center. Written informed consent for participation was not required for this study in accordance with the national legislation and the institutional requirements.

## Author contributions

LZ, YL, and KX conceived the central ideas of the study. YF and ZWang collected the data. LZ wrote the first draft of the manuscript. YL and KX revised the paper, worked on the English, and drafted the final version of the manuscript. ZWei and YZ revised the paper. All authors contributed to the article and approved the submitted version.

## Supplementary material

Supplementary Material 1 The variables contained in the most streamlined model screened by the lasso regression model.

Supplementary Material 2 Blood pressure effect on the prognosis of sepsis patients with acute kidney injury.
