# Supplementary material for: The blood pressure targets in sepsis patients with acute kidney injury: An observational cohort study of multiple ICUs
Source: Front Immunol. 2022 Dec 15;13:1060612. doi: 10.3389/fimmu.2022.1060612 (PMC9797512; doi:10.3389/fimmu.2022.1060612)
Supplement: Supplementary file 3 [file DataSheet_3.docx]

| **Supplementary material 2** Blood pressure effect on the prognosis of sepsis patients with acute kidney injury | | | | | | |
| --- | --- | --- | --- | --- | --- | --- |
|  | | | | | | |
| Patients without hypertension disease | | | | | | |
|  | MAP (64-73)mmHg  n=672 | Non-MAP(64-73)mmHg  n=3574 | P | DBP(50-60)mmHg  n= 800 | Non-DBP(50-60) mmHg  n=3446 | P |
| Urine output (ml) | 777.50[150.00, 1757.00] | 950.00[300.00, 1757.00] | 0.007 | 885.00[175.00, 1762.75] | 929.50[300.00, 1757.00] | 0.145 |
| Creatinine (mg/dL) | 1.83 [1.20, 2.80] | 1.90 [1.30, 3.00] | 0.032 | 1.80 [1.20, 2.80] | 1.90 [1.30, 3.00] | 0.086 |
| Blood urea nitrogen (mg/dL) | 35.00 [22.00, 56.00] | 37.00 [24.00, 58.00] | 0.032 | 35.00[22.00, 54.00] | 38.00 [24.00, 58.75] | 0.008 |
| RRT, n (%) | 198 ( 29.5) | 757 ( 21.2) | <0.001 | 228 ( 28.5) | 727 ( 21.1) | <0.001 |
| Mechanical ventilation, (n(%)) | 460 ( 68.5) | 2521 ( 70.5) | 0.299 | 549 ( 68.6) | 2432 ( 70.6) | <0.001 |
| Arrhythmia, n (%) | 121 ( 18.0) | 1541 ( 43.1) | <0.001 | 288 ( 36.0) | 1374 ( 39.9) | 0.048 |
| Length of icu stays, days | 3.90 [1.74, 9.28] | 3.40 [1.80, 7.60] | 0.103 | 3.84 [1.82, 9.21] | 3.40 [1.80, 7.54] | 0.080 |
| Length of hospital stays, days | 11.00 [6.00, 21.02] | 11.50 [6.20, 20.60] | 0.909 | 10.95 [6.10, 20.72] | 11.60 [6.20, 20.70] | 0.847 |
| Hospital mortality, (n (%) | 194 ( 28.9) | 1090 ( 30.5) | 0.425 | 229 ( 28.6) | 1055 ( 30.6) | 0.289 |
| Patients with hypertension disease | | | | | | |
|  | MAP (70-80) mmHg  n=115 | Non-MAP (70-80) mmHg  n=1472 | P | DBP(55-62)mmHg  n=482 | Non-DBP(55-62)mmHg  N=1105 | P |
| Urine output(mL) | 575.00[137.50, 1757.00] | 1224.00[575.00, 1910.75] | <0.001 | 1227.50 [491.75, 1895.50] | 1197.00 [525.00, 1900.00] | 0.793 |
| Creatinine (mg/dL) | 1.40[0.92, 2.05] | 1.60 [1.10, 2.92] | 0.005 | 1.40 [1.02, 2.10] | 1.70 [1.10, 3.40] | <0.001 |
| Blood urea nitrogen (mg/dL) | 28.00 [15.50, 47.50] | 33.00 [21.00, 59.00] | 0.001 | 28.00[19.00, 43.00] | 36.00 [21.00, 68.00] | <0.001 |
| RRT,n(%) | 40 ( 34.8) | 216 ( 14.7) | <0.001 | 87 ( 18.0) | 169 ( 15.3) | 0.194 |
| Mechanical ventilation, (n(%)) | 78 ( 67.8) | 1078 ( 73.2) | 0.251 | 362 ( 75.1) | 794 ( 71.9) | 0.202 |
| Arrhythmia, n (%) | 1 ( 0.9) | 79 ( 5.4) | 0.057 | 1 ( 0.2) | 79 ( 7.1) | <0.001 |
| Length of ICU stays, days | 4.70 [2.23, 10.65] | 3.24 [1.64, 7.14] | 0.005 | 3.48 [1.83, 7.74] | 3.27 [1.64, 7.15] | 0.202 |
| Length of hospital stays, days | 13.10 [7.70, 22.15] | 11.70 [6.70, 20.50] | 0.164 | 12.40 [6.80, 20.67] | 11.60 [6.90, 20.80] | 0.374 |
| Hospital mortality, (n(%) | 24 ( 20.9) | 317 ( 21.5) | 0.960 | 92 ( 19.1) | 249 ( 22.5) | 0.141 |

RRT: renal replacement therapy; ICU: intensive care unit.
